# Adenomatous polyposis coli is required for early events in the normal growth and differentiation of the developing cerebral cortex

**DOI:** 10.1186/1749-8104-4-3

**Published:** 2009-01-16

**Authors:** Uladzislau Ivaniutsin, Yijing Chen, John O Mason, David J Price, Thomas Pratt

**Affiliations:** 1 Genes and Development Group, Centre for Integrative Physiology, School of Biomedical Sciences, The University of Edinburgh, Hugh Robson Building, George Square, Edinburgh, UK

## Abstract

**Background:**

Adenomatous polyposis coli (Apc) is a large multifunctional protein known to be important for Wnt/β-catenin signalling, cytoskeletal dynamics, and cell polarity. In the developing cerebral cortex, *Apc *is expressed in proliferating cells and its expression increases as cells migrate to the cortical plate. We examined the consequences of loss of Apc function for the early development of the cerebral cortex.

**Results:**

We used *Emx1*^*Cre *^to inactivate *Apc *specifically in proliferating cerebral cortical cells and their descendents starting from embryonic day 9.5. We observed reduction in the size of the mutant cerebral cortex, disruption to its organisation, and changes in the molecular identity of its cells. Loss of Apc leads to a decrease in the size of the proliferative pool, disrupted interkinetic nuclear migration, and increased apoptosis. β-Catenin, pericentrin, and N-cadherin proteins no longer adopt their normal high concentration at the apical surface of the cerebral cortical ventricular zone, indicating that cell polarity is disrupted. Consistent with enhanced Wnt/β-catenin signalling resulting from loss of Apc we found increased levels of TCF/LEF-dependent transcription and expression of endogenous Wnt/β-catenin target genes (*Axin2 *(*conductin*), *Lef1*, and *c-myc*) in the mutant cerebral cortex. In the *Apc *mutant cerebral cortex the expression of transcription factors *Foxg1*, *Pax6*, *Tbr1*, and *Tbr2 *is drastically reduced compared to normal and many cells ectopically express *Pax3*, *Wnt1*, and *Wt1 *(but not *Wnt2b*, *Wnt8b*, *Ptc*, *Gli1*, *Mash1*, *Olig2*, or *Islet1*). This indicates that loss of Apc function causes cerebral cortical cells to lose their normal identity and redirect to fates normally found in more posterior-dorsal regions of the central nervous system.

**Conclusion:**

Apc is required for multiple aspects of early cerebral cortical development, including the regulation of cell number, interkinetic nuclear migration, cell polarity, and cell type specification.

## Background

In the mouse, the cerebral cortex develops from anterior neuroepithelium starting around half-way through gestation at embryonic day (E)9.5. During its early development the cerebral cortex is divided into the ventricular zone, the subventricular zone, the intermediate zone, and the cortical plate. Cortical progenitors have radial processes that span the depth of the cerebral cortex and have endfeet at its apical and basal surfaces. Cell division occurs in the ventricular and subventricular zones and daughter cells either undergo more cell divisions or exit the cell cycle and migrate through the intermediate zone to the cortical plate where they undergo further differentiation and project axons into the intermediate zone. Eventually, different regions of the cerebral cortex exhibit distinct cytoarchitecture and function and it is believed that the areal expression of secreted morphogens and transcription factors contribute to setting this up during development. For reviews of these processes, see [1-5].

*APC *(*Adenomatous polyposis coli*) was originally identified as a tumour suppressor gene mutated in familial adenomatous polyposis, an autosomal dominant condition with predisposition to colorectal cancers [6] and brain tumours [7]. Mutations in *APC *have also been linked to a case of mental retardation [8]. Mutations in *Apc *(the murine homologue of *APC*) have been linked to intestinal neoplasia in mice [9]. Many genes whose dysfunction is associated with tumour formation have important functions in the normal development of the nervous system [10]. Apc is now known to be involved in regulating a variety of cellular processes, including mitosis, cytoskeletal dynamics, axonogenesis, cell polarity and apoptosis [11-16]. Apc is central to the Wnt signalling pathway, in which it mediates the destruction of cytoplasmic β-catenin protein [17] unless the cell receives a Wnt signal resulting in the stabilisation of β-catenin and its translocation to the nucleus, where it co-operates with TCF/LEF to activate the transcription of target genes. Wnt/β-catenin signalling has diverse roles in central nervous system development [18]. Given the various functions of Apc, there are many ways in which Apc might influence the development of cerebral cortical cells.

It has previously been shown that *Apc *mRNA is widely expressed throughout the developing brain. In the cerebral cortex, proliferating cells express *Apc *mRNA and its expression levels increase as cells migrate to the cortical plate and differentiate [19]. Apc is known to be a regulator of β-catenin activity and the levels of β-catenin signalling change as cells stop dividing and move from the ventricular zone, through the intermediate zone, and into the cortical plate [20]. These observations raise the possibility that Apc protein might be important for proliferation and/or the subsequent differentiation of cells in the cerebral cortex. Apc has also been implicated in cerebral cortical development via its interaction with the cytoskeletal protein Lis1 [21], indicating that Apc has functions not directly related to Wnt/β-catenin signalling.

In this study we test the hypothesis that Apc is important for early events in the formation of the cerebral cortex using a Cre/LoxP strategy. We use a floxed *Apc *allele, *Apc*^*580S *^[22], in which Cre-induced recombination leads to deletion of *Apc *exon 14 and a frameshift at codon 580 that has been shown to disrupt the function of Apc [23]. We used the *Emx1*^*Cre *^allele [24] to produce this mutation of *Apc *specifically in cells of the developing cerebral cortex starting at E9.5. Cerebral cortex depleted of Apc exhibits reduced size, a massive disorganisation of cell types, and a profound alteration in the identity of many of its cells. These defects coincide with increased levels of nuclear β-catenin and up-regulation of Wnt/β-catenin target genes as well as alterations to the organisation of the cytoskeleton and cell polarity.

## Materials and methods

### Animals

Mice harbouring various combinations of the following alleles were used in this study: *Apc*^*580S *^[22]; *Emx1*^*Cre *^[24]; *BAT-gal *[25]; *Rosa26R *[26]. Timed matings between *Emx1*^*Cre*/*Cre*^*Apc*^*580S*/+ ^or *Emx1*^*Cre*/+^*Apc*^*580S*/+ ^and *Emx1*^+/+^*Apc*^*580S*/*580S *^animals were used to generate experimental embryos with the plug day designated as E0.5. Throughout this manuscript embryos are designated 'control' (*Emx1*^*Cre*/+^*Apc*^*580S*/+^) or 'mutant' (*Emx1*^*Cre*/+^*Apc*^*580S*/*580S*^). Other genotypes were not used.

### Injection of S-phase tracers and estimation of proliferative pool and cell cycle kinetics

For estimation of the proliferative pool size, cumulative bromodeoxyuridine (BrdU) labelling was performed by injecting BrdU (200 μl of 100 μg/ml (in 0.9% NaCl) (Sigma St. Louis, MO, USA) intra-peritoneally to pregnant females every 2 hours over a 12-hour period. Embryos were collected after 12 hours from the first injection. For estimation of cell cycle kinetics, double labelling experiments were done by intra-peritoneal injection of iododeoxyuridine (IdU; 200 μl of 100 μg/ml in 0.9% NaCl (Sigma)) and BrdU (200 μl of 100 μg/ml (in 0.9% NaCl) (Sigma)) 1.5 hours later. Animals were sacrificed 30 minutes after BrdU injection following a protocol previously described [27]. Coronal sections of cerebral cortex were immunostained for BrdU and IdU and cell cycle parameters were calculated as described [27].

### Genotyping

The following pairs of primers were used for PCR genotying of genomic DNA extracted from biopsies: *Apc *forward 5'-CACTCAAAACGCTTTTGAGGGTTGAAT-3', reverse GTTCTGTATCATGGAAAGATAGGTGGT (product size: 226 base-pairs (wild type allele) 314 base-pairs (580S floxed allele); *Emx1 *forward 5'-TGGCCCAACTCGGTGTTAGG-3', reverse 5'-CCACCAAGGACTCTATGGTG-3' (product size 260 base-pairs); *Cre *forward 5'-ACCTGATGGACATGTTCAGGGATC-3', reverse 5'-TCCGGTTATTCAACTTGCACCATG-3' (product size 108 base-pairs); *LacZ *forward 5'-CGAAATCCCGAATCTCTATCGTGC-3', reverse 5'-GATCATCGGTCAGACGATTCATTGG-3' (product size 400 base-pairs).

### *In situ *hybridisation

Embryonic heads (E13.5) were submerged in a solution of 4% paraformaldehyde, 0.1% Tween20 in phosphate buffered saline (PBS) pH9.5 for 8 to 20 hours at 4°C on a rocking platform, embedded in wax, and cut into sections 10 μm thick. *In situ *hybridisations for *Axin2*, *Lef1*, *Wnt2b*, *Wnt8b*, *Gli1*, and *Ptc *transcripts were performed as described previously [28] using digoxygenin-labelled antisense riboprobes.

### Immunohistochemistry

Embryonic heads (E13.5 to E15.5), or whole embryos (E12.5 and earlier) were submerged in a solution of 4% paraformaldehyde in PBS for 8 to 20 hours at 4°C on a rocking platform. Heads were either: embedded in wax and cut into sections 10 μm thick; frozen and cut into sections 10 μm thick; or embedded in agarose and cut into vibratome sections 100 μm thick. Wax sections were dewaxed in xylene and rehydrated through a series of solutions of descending ethanol concentration to water. Sections were microwaved to unmask the antigen epitope. Chromogenic visualization was done with Envision^+ ^Kit (Dako, Ely, Cambridgeshire, UK). In some cases sections were counterstained with Harris-haematoxylin (Thermo Electron Corporation, Cheshire, UK). For immunofluorescence, following incubation with the primary antibody, sections were incubated with species-specific secondary antibodies conjugated to fluorescent molecules at a 1:200 dilution: goat anti-mouse or goat anti-rabbit Alexafluor-488 or Alexafluor-568 (Invitrogen, Paisley, UK). For detection of IdU/BrdU, a mouse anti-BrdU antibody that recognises both IdU and BrdU was used (clone B44, 1:100 in blocking solution; Becton Dickinson Oxford, UK). Rat anti-BrdU antibody (clone BU1/75, 1:100; Abcam, Cambridge, UK) was used to detect BrdU but not IdU. In case of double labelling, goat anti-mouse highly cross-absorbed antibodies were used to prevent cross-reactivity. Nuclei were counterstained using the DNA dye TO-PRO-3 iodide (Invitrogen) at 1 μM. Sections were mounted under coverslips using Mowiol to prevent fading of fluorescence. The following additional primary antibodies were used at the dilutions stated: Apc(C-20) rabbit, sc-896, 1:400 (Santa Cruz Biotechnology, Santa Cruz, CA, USA) immunohistochemistry on vibratome sections and dissociated cells); Apc (ab15270), rabbit polyclonal, 1:100 (Abcam; immunohistochemistry on wax sections); β-catenin, mouse, 610154, 1:200 (BD Biosciences UK, Oxford, UK) beta-III-tubulin, mouse, Tuj1, 1:800 (Sigma); P21, rabbit, SX118, 1:100 (BD Biosciences); Pax3, mouse, Pax3, 1:400 (DSHB, University of Iowa, Iowa City, USA); Foxg1, rabbit rabbit polyclonal antibody described in [29], 1:500; C-myc, mouse, 9E10, 1:200 (Roche Diagnostics GmbH, Mannheim, Germany); phosphohistone H3 (PH3), rabbit, H9908, 1:400 (Sigma); WT1, mouse, 6F-H2, 1:1000 (Dako); N-cadherin, mouse, 610920, 1:200 (BD Biosciences); Pericentrin, rabbit, PRB-432C, 1:400 (Covance Emeryville, CA, USA); Tbr1 and Tbr2, rabbit polyclonal antibodies described in [30], 1:500; Olig2, rabbit polyclonal antibodies described in [31], 1:10,000; Mash1 mouse monoclonal 24B7.2d11, 1:100 (BD Biosciences); Islet1, mouse 40.2D6, 1:100 (DSHB); Pax6, mouse, Pax6, 1:400 (DSHB).  

### Staining for bacterial LacZ (β-galactosidase)

Embryos were dissected in ice cold PBS and fixed with shaking at 4°C for 1 hour in LacZ fixative (PBS containing: paraformaldehyde (4%); NP40 (0.02%), sodium deoxycholate (0.01%); EGTA (5 mM); and MgCl2 (2 mM)). LacZ staining was as described previously [25].

### Detection of apoptosis by TUNEL

To detect apoptotic cells, terminal deoxynucleotidyl nick end labelling (TUNEL) was performed according to the supplier's protocol (Roche). Total numbers of TUNEL positive cells were counted in sections of E13.5 control and mutant embryos.

### Primary cell culture and FACS analysis

The telencephalon was isolated and dissected in ice-cold oxygenated Earle's buffered salts solution. The cerebral cortex was separated from the ventral telencephalon. Telencephalic tissue was dissociated using Papain dissociation system kit (Worthington Biochemical Corporation, Lakewood, NJ, USA) according to the supplier's protocol. Cells were resuspended in serum-free medium. Dissociated cells were then either: embedded in collagen [32] and cultured for 4 hours, after which time they were fixed and processed for Apc immunohistochemistry; or subjected to fluorescence activated cell scanning (FACS) analysis following fixation in -20°C 70% ethanol for ≥ 2 hours on ice and resuspension in PBS containing 0.05 mg/ml of propidium iodide (Sigma) and 0.5 mg/ml of RNAseA (Roche). FACS analysis was performed on FACSCalibur (BD Biosciences), which runs CellQuest software.

### Microscopy

Slides were photographed using a Leica DMLB upright compound microscope connected to a Leica DSC480 digital camera with Leica IM50 image management software. Fluorescent staining was imaged using a Leica TCS NT confocal system with associated software. Alexafluor-488 staining was collected in the FITC (green) channel, Alexafluor-568 in the TRITC (red) channel and TO-PRO-3 in the Cy3 (far-red, pseudo-coloured blue) channel.

### Statistical analysis and graph plotting

Sigmastat (Systat Software Inc., Richmond, CA, USA) was used for data analysis. Excel (Microsoft) was used to plot data.

### cDNA synthesis and quantitative reverse transcriptase PCR

RNA was extracted from the cerebral cortical tissue at E12.5, E13.5, and E15.5 using RNEasy mini kit (Qiagen, Crawley, Sussex, UK) followed by treatment with DNAse (Roche) to eliminate genomic DNA. The SuperScript III First-Strand Synthesis SuperMix kit (Invitrogen) was used to synthesize cDNA from RNA samples. The procedure was performed according to the supplier's protocol using random hexamers. The following primers were used for quantitative reverse transcriptase PCR (qRT-PCR): *Apc *exon 4 forward 5'-CCGTTCAGGAGAATGCAGTC-3', reverse 5'-TGCCGTCTTGTCATGTCTGT-3'; *Apc *exon 14 forward 5'-GTGTCCAGCTTGATAGCTAC-3', reverse 5'-CAAGGCTTCCTGGTCTTTAG-3'; *Wnt1 *forward 5'-ATACGACCCCGTTTCTGCTG-3', reverse 5'-TTCCACTCCCTCACCTCAAAGC-3'; *Axin2 *forward 5'-ACAGTAGCGTAGATGGAGTC-3', reverse 5'-CTGTGGAACCTGCTGCCTTC-3'; *Wnt8b *forward 5'-AAGGCTTACCTGGTCTACTC-3', reverse 5'-CAGAGCTGATGGCGTGCACA-3'; *GAPDH *5'-GGGTGTGAACCACGAGAAAT-3' and 5'-CCTTCCACAATGCCAAAGTT-3'. qRT-PCR was performed using Qiagen Quantitect SYBR green PCR kit and a DNA Engine Opticon continuous fluorescence detector (Genetic Research Instrumentation, Rayne, Essex, UK) The abundance of each transcript in the original RNA sample was extrapolated from PCR reaction kinetics using Opticon software. Transcript levels are expressed relative to GAPDH.

## Results

### Apc expression in developing wild-type and mutant cerebral cortex

**Figure 1:**
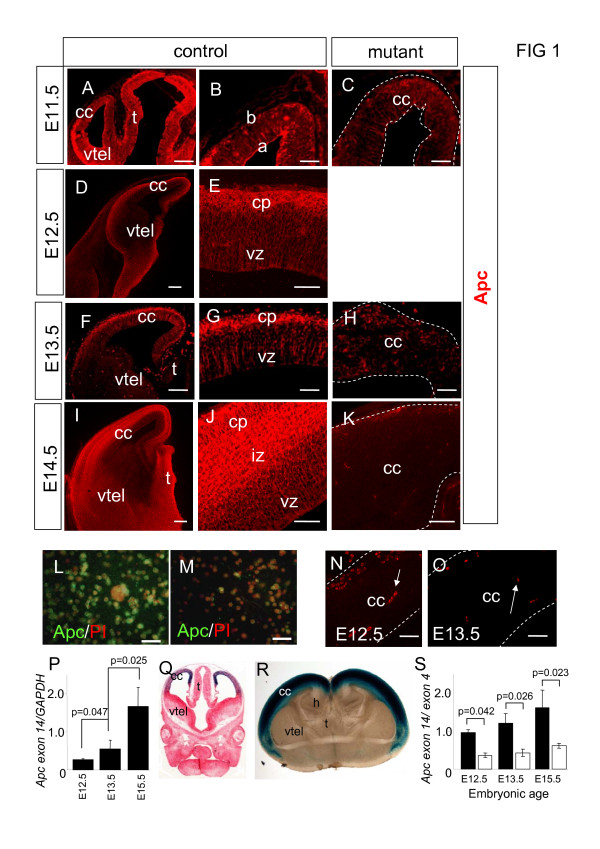
**Apc/*Apc *expression in the cerebral cortex during normal development and following Cre-mediated deletion of *Apc***. **(A, D, F, I) **Immunohistochemistry showing Apc expression in the developing forebrain of control embryos at embryonic day (E)11.5 (A), E12.5 (D), E13.5 (F), and E14.5 (I). **(B, E, G, J) **Higher power magnification of the control cerebral cortex. (B) At E11.5, Apc is expressed throughout the cerebral cortex (cc) with prominent expression at its apical (a) surface compared to the basal (b) surface. (E) At E12.5, Apc is expressed throughout the cerebral cortex in the ventricular zone (vz) and the newly formed cortical plate (cp) where its expression levels are highest. (G, J) At E13.5 (G) and E14.5 (J), Apc is expressed throughout the cerebral cortex c. Apc expression extends from the apical surface of the ventricular zone to the pial edge with the highest levels of expression in the cortical plate and intermediate zone (iz). **(C, H, K) **Apc expression is reduced in the cerebral cortex of mutant embryos compared to controls at E11.5 (C), E13.5 (H) and E14.5 (K). The white dotted lines demarcate the mutant cerebral cortex. **(L, M) **Apc immunocytochemistry of dissociated E13.5 control (L) and mutant (M) cells showing that Apc expression is reduced in the mutant. Nuclei stained red with Propidium (PI). **(N, O) **Controls showing that the secondary antibody used to detect Apc does not stain neural cells at E12.5 (N) and E13.5 (O), although blood vessels (arrows) are strongly fluorescent. **(P)***Apc *transcript levels increase during normal corticogenesis from E12.5 to E13.5 and E13.5 to E15.5. Histogram shows levels of *Apc *relative to GAPDH in mRNA extracted from cerebral cortex at E12.5, E13.5 and E15.5. Black bars are control and white bars are mutant. Student's *t*-test *p*-values are indicated above the bars (comparison between all groups ANOVA *p *= 0.002). **(Q, R) **LacZ staining (blue) of E12.5 (Q) and E16.5 (R) *Emx1*^*Cre*/+^; *R26R *forebrain, confirming the ability of Cre recombinase to recombine a floxed R26R reporter allele and turn on LacZ expression in the cerebral cortex. **(S) **Mutant cerebral cortex has significantly reduced levels of wild-type *Apc *transcripts compared to controls. Histogram shows the ratio of transcripts containing exon 14 (deleted following Cre-mediated recombination of *Apc*^*580S *^allele) to exon 4 at E12.5, E13.5, and E15.5. Student's *t*-test *p*-values are indicated above the bars (comparison between all groups ANOVA *p *= 0.001). There is no significant difference in the ratio obtained from control embryos at the ages examined (ANOVA *p *= 0.390). (D, E, I, J, K, O) Vibratome sections; (A-C, F-H, N) wax sections; (L, M) dissociated cells embedded in collagen. Additional abbreviations: h, hippocampus; t, thalamus; vtel, ventral telencephalon. All sections are coronal and dorsal is up. Scale bars: (A, D, F, I) 200 μm; (B, C, E, G, H, J, K, N, O) 50 μm; (L, M) 25 μm. Error bars in (P, S) are standard error of the mean.

During normal development, *Apc *mRNA is expressed throughout the cerebral cortex, with the highest levels of expression in post-mitotic cells that have migrated from the ventricular zone [19]. Here we extended this study by examining the distribution of Apc protein using immunohistochemistry. Apc is widely expressed in the forebrain, with particularly high levels in the cerebral cortex (Figure 1A, D, F, I). As development proceeds from E11.5 to E14.5, Apc is expressed throughout the proliferative ventricular zone from its apical surface. At E11.5, Apc expression is particularly prominent at the apical surface of the ventricular zone (Figure 1B) with a few high expressing cells populating the basal surface. As cerebral cortex development proceeds from E12.5 to E14.5, Apc continues to be expressed in the ventricular zone but the highest levels of expression are now seen in post-mitotic cells in the cortical plate (Figure 1E, G, J) and in the intermediate zone, which contains cells migrating to the cortical plate and axons projected by post-mitotic cortical plate cells after E13.5 (Figure 1J). qRT-PCR results show that there is a dramatic increase in the levels of *Apc *transcripts between E12.5 and E15.5 (Figure 1P). This increase coincides with the increasing proportion of high-expressing post-mitotic neurons present in the cerebral cortex with increasing age.

We used Cre recombinase driven by the endogenous *Emx1 *promoter [24,33] to inactivate *Apc *expression from a floxed *Apc *allele in neural cells in the developing cerebral cortex starting at E9.5 when the cerebral cortex is starting to form. LacZ staining of embryos harbouring *Emx1*^*Cre *^and *R26R *reporter alleles[26] confirms the ability of Cre recombinase to recombine the floxed-stop *LacZ *reporter allele in the cerebral cortex of these embryos (Figure 1Q, R). *Emx1 *is not expressed in the most dorsal medial telencephalon (the cortical hem region) [33,34] and this region escapes Cre-mediated recombination (Figure 1Q, R). The efficiency of Cre recombination (judged from the intensity of LacZ staining) appears to diminish towards the lateral edges of the cerebral cortex (Figure 1Q, R) and, for this reason, we concentrated our subsequent analysis of the *Apc *mutant phenotype on the central portion of the cerebral cortex. We used the *Apc*^*580S *^allele in which exon 14 of *Apc *is flanked by *LoxP *sites [22]. Transcripts produced from the *Apc*^*580S *^allele following Cre-mediated recombination lack exon 14, causing a frameshift, and translation of these transcripts is predicted to produce non-functional Apc. In order to confirm that *Apc *levels were reduced in the cerebral cortex of our mutant, we performed qRT-PCR using primers to exon 4 (present in transcripts produced from the floxed *Apc *both before and after Cre-mediated recombination) and exon 14 (only present in transcripts produced from un-recombined *Apc*) on RNA extracted from dorsal telencephalon of control and mutant embryos at E12.5, E13.5, and E15.5. The ratio of *Apc *transcripts containing exon 14 to those containing exon 4 provides a measure of the efficiency with which wild-type *Apc *transcripts were eliminated from the mutant cortex. At all ages examined we found that the exon 14:exon 4 ratio was reduced in the mutant compared to the controls (Figure 1S), indicating successful reduction in the levels of wild-type *Apc *transcripts in the mutant. The low levels of wild-type transcripts in RNA samples from the mutant cortex might reflect subpopulations of cells within the cerebral cortex that escaped recombination in the mutant (for example blood vessels) and/or persistence of wild-type *Apc *transcripts after recombination of the genomic locus. Comparison of Apc immunofluorescence on sections and collagen-embedded dissociated cells from control (Figure 1B, E, G, J, L) and mutant (Figure 1C, H, K, M) cerebral cortex showed that the intensity of immunostaining was much lower in the mutant cells, indicating successful depletion of Apc protein. The intense spots of Apc staining in Figure 1H, K correspond to non-specific staining of blood vessels by the fluorescent secondary antibody (blood vessels are strongly stained in controls reacted with the fluorescent secondary antibody alone; Figure 1N, O). Apc immunohistochemistry on dissociated cells indicates that some cells in the mutant cerebral cortex retain Apc protein, albeit at reduced levels to that seen in control cerebral cortex (compare Figure 1L to 1M). In summary, mutant embryos are unable to express *Apc *mRNA or Apc protein at the same levels as their wild-type counterparts and do not upregulate *Apc*/Apc expression as occurs during normal cerebral cortical development.

### Neuronal differentiation is disrupted when Apc is lost from cerebral cortex

**Figure 2:**
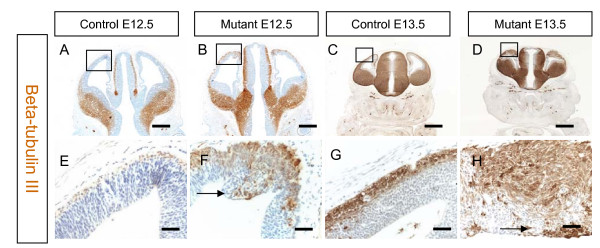
**Immunostaining for beta-tubulin III (Tuj1) at embryonic day (E)12.5 and E13.5 in the cerebral cortex of control and mutant embryos**. **(A, B, E, F) **E12.5. (A, E) At E12.5, Tuj1 staining is restricted to cells of the preplate of the control cerebral cortex. (B, F) The mutant has a broader Tuj1 expression pattern with positive cells located in the ventricular surface (F, black arrow). **(C, D, G, H) **There is a similar staining pattern at E13.5. (C, G) The control has Tuj1 positive cell in the preplate only whereas (D, H) the mutant has more Tuj1 staining with positive cells located in at the ventricular surface (H, black arrow). Boxed areas in (A-D) are shown at higher magnification in (E-H). All sections are coronal and dorsal is up. Scale bars: (A, B) 200 μm; (C, D) 400 Mm; (E-H) 50 μm.

Apc is a multifunctional protein with potential roles in several aspects of cerebral cortical development. We first set out to determine the consequence of loss of Apc function on the organisation and proliferation of cerebral cortical cells. β-Tubulin III (Tuj1) is a neural form of β-tubulin, which is expressed by post-mitotic neurons [35]. β-Tubulin immunostaining of control and mutant cerebral cortex at E12.5 and E13.5 revealed that the mutant cerebral cortex is disorganised. In controls, β-tubulin is located in post-mitotic cells in the cortical plate (Figure 2A, C, E, G). In mutants there are β-tubulin stained cells in the cortical plate but stained cells are also found in close proximity to the ventricle (Figure 2B, D, F, H). These results show that while depleting Apc in developing cerebral cortex does not prevent the production of post-mitotic neurons, it does disrupt their ability to occupy their normal location in the developing cortical plate. This could reflect precocious differentiation, defective radial migration, or a combination of the two.

### Loss of Apc leads to a reduction in the proliferative pool

The small size of the mutant cerebral cortex prompted us to ask whether there was a defect in proliferation. Continuous cumulative injection of the thymidine analogue BrdU labels all cells that are undergoing S-phase during the pulse [36].

**Figure 3:**
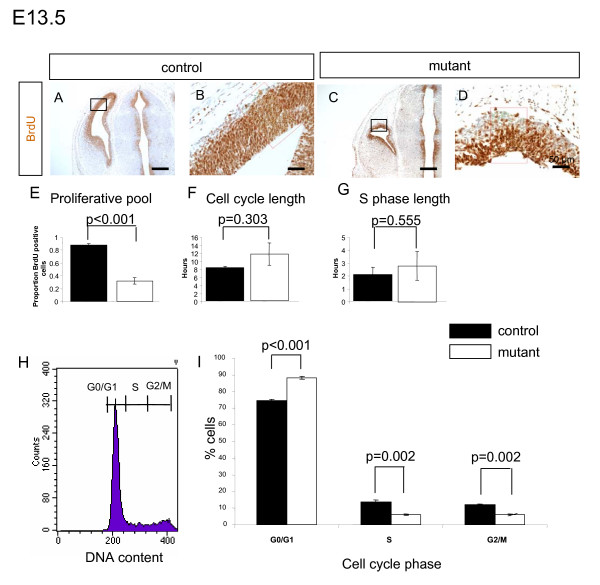
**Reduced proliferative pool in mutant embryos compared to the control at embryonic day (E)13.5**. **(A-D) **Control (A, B) or mutant embryos (C, D) were pulsed with bromodeoxyuridine (BrdU) for 12 hours to label all cells that had passed through S-phase during the pulse. (A, C) Low power images with boxed areas indicating location of higher magnification (B, D) images. **(E) **Quantification of the proportion of BrdU-positive cells as a proportion of total cells. The proportion of BrdU-positive cells is significantly lower in the mutant. **(F, G) **There are no significant differences in cell cycle (F) or S-phase (G) length between mutant and control embyos. **(H, I) **FACS analysis of DNA content of E13.5 control and mutant cerebral cortical cells. (H) A histogram of dissociated cells from control cerebral cortex indicating populations in G0/G1, S, and M-phases of the cell cycle sorted by their DNA content. (I) The proportion of cells in each phase of the cell cycle, calculated from histograms like those shown in (H), in control and mutant cerebral cortex. The mutant has significantly more cells in G0/G1 and significantly fewer cells in S/G2 than the mutant, consistent with a reduction in the population of proliferating cells. All sections are coronal and dorsal is up. Scale bars: (A, C) 400 μm; (B, D) 50 μm. Error bars in (E, F, G, I) are standard error of the mean with Student's *t*-test *p*-values for control versus mutant comparison indicated above bars.

Immunostaining of the cerebral cortex of E13.5 embryos continuously labelled with BrdU for 12 hours revealed that the cerebral cortex of the mutant contains a larger proportion of cells that have not incorporated BrdU than the control (compare Figure 3A, B to 3C, D). Quantification of the relative size of the BrdU-incorporating population disclosed a significant reduction of BrdU positive cells in the mutant cerebral cortex compared to the control (Figure 3E). A similar result was obtained for E12.5 (not shown). This reduction in the size of the proliferative pool could reflect alterations in the length of the phases of the cell cycle, so we investigated these parameters using the two nucleotide analogues BrdU and IdU [27]. This technique allows us to determine both Tc (cell cycle length) and Ts (S-phase length) from a single experiment. This showed that although the number of proliferating cells is lower in mutant brains, there is no significant difference in the length of either the cell cycle (Figure 3F) or S-phase (Figure 3G) between mutant and control embryos in the poliferating cells that remain. We used FACS analysis to determine the proportion of proliferating cells that were in the G0/G1, S, or G2/M stages of the cell cycle. Cell sorting was performed on dissociated cells taken from E13.5 control and mutant cerebral cortex and stained with the DNA binding dye propidium iodide to generate histograms of DNA content (example shown in Figure 3H). These histograms were used to calculate the cell populations in different phases of the cell cycle. The S and G2/M populations in the mutant were reduced to 50% of numbers in control embryos (Figure 3I). There was a corresponding increase in the size of the G0/G1 population in the mutant (Figure 3I). Loss of Apc therefore reduces the number of proliferating cells by about three-fold but does not significantly affect the cell-cycle kinetics of the remaining proliferating cells. The reduction in the size of the proliferative pool could be caused by cell-cycle arrest, premature differentiation, or death.

### Loss of Apc causes increased apoptosis

**Figure 4:**
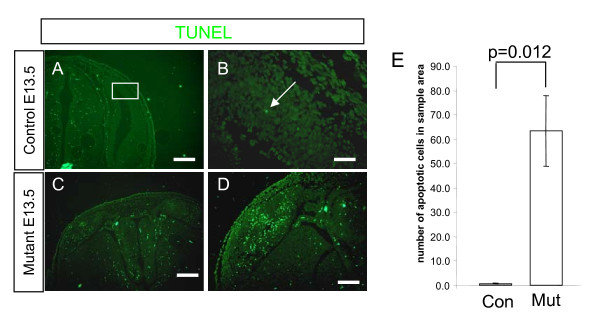
**Apoptosis in the cerebral cortex is increased in mutant embryos at embryonic day (E)13.5**. **(A, B) **A few apoptotic cells were detected by TUNEL staining in the control. (B) A higher magnification of the boxed area in (A) with white arrow indicating TUNEL^+ ^cell (green staining). **(C, D) **Examples of the mutant showing increased apoptosis in the cerebral cortex **(E) **Quantification of apoptotic nuclei in sections of control and mutant cerebral cortex shows a large increase in apoptosis in the mutant. Student's *t*-test *p*-values are indicated above bars. Error bars are standard error of the mean. All sections are coronal and dorsal is up. Scale bars: (A, C) 400 μm; (B) 50 μm; (D) 200 μm.

The reduced size of the mutant cortex could be contributed to by increased apoptosis and we next examined this possibility. Both pro-apoptotic and anti-apoptotic activities of *Apc *mutations have been described [37-39]. Investigation of apoptosis by TUNEL staining revealed that whereas there are very few apoptotic cells detected in the control cerebral cortex at E13.5 (Figure 4A, B), there are many apoptotic cells detected in the cerebral cortex of the mutant at this stage (Figure 4C, D). Counts of apoptotic cells in sections of cerebral cortex confirmed a massive and significant increase in cell death in the mutant compared to the control cerebral cortex (Figure 4E).

### Disruption to spatial organisation of the cell cycle and cell polarity

**Figure 5:**
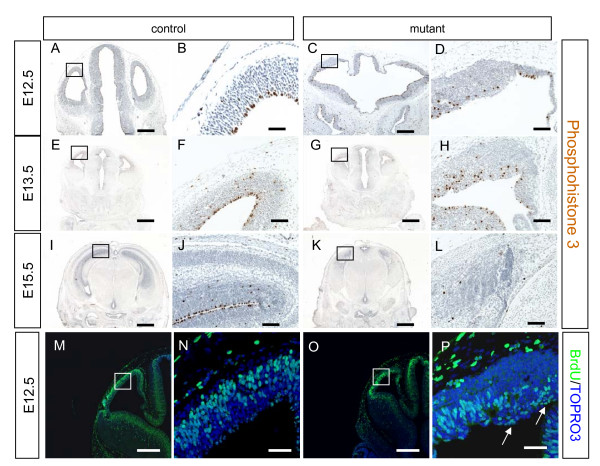
**Disrupted interkinetic  nuclear  migration in mutant cerebral cortex progenitor cells**. **(A-L)** Immunostaining for phosphohistoneH3 (PH3; brown) in the telencephalon of control and mutant embryos at embryonic day (E)12.5, E13.5 and E15.5. At E12.5, most of the PH3-positive cells in the control cerebral cortex are located at the ventricular surface (B). Most PH3-positive cells do not touch the ventricular surface of the mutant (D). At E13.5, the control has most of the PH3-positive cells at the ventricular surface (F), but most PH3-positive cells in the mutant are located away from the ventricular surface (H). At E15.5, the control has PH3-positive cells on the ventricular surface and singular cells in the ventricular zone and subventricular zone (J), while expression of PH3 in the mutant is decreased and positive cells are scattered in the cerebral cortex (L). Blue staining is counterstaining with haematoxylin. (B, D, F, H, J, L) Higher magnification images of the boxed areas in (A, C, E, G, I, K), respectively. **(M-P) **Bromodeoxyuridine (BrdU) incorporation in embryos sacrificed immediately after a 30 minute pulse of BrdU labels cells in S-phase. In controls (M, N), BrdU labelled cells are all located away from the ventricular surface whereas in the mutant (O, P) many BrdU positive cells are located at the ventricular surface. (N, P) Higher magnification images of the boxed areas in (M, O), respectively. All sections are coronal and dorsal is up. Scale bars: (A, C, M, O) 200 μm; (B, D) 25 μm; (E, G, I, K) 400 μm; (F, H, N, P) 50 μm.

During normal cerebral cortical development, neural progenitors undergo interkinetic nuclear migration during the cell cycle such that cell nuclei undergo mitosis (M-phase) when they are located at the ventricular surface and then move deeper into the ventricular zone where they perform DNA synthesis (S-phase) (for example, see [40]). PH3 is a M-phase specific protein [41], which allows us to identify cells that are undergoing mitosis. PH3 immunostaining of control cerebral cortex at E12.5, E13.5, and E15.5 (Figure 5A, B, E, F, I, J) shows most mitotic cells lining up along the ventricular surface. In contrast, in mutants over the same age range (Figure 5C, D, G, H, K, L) nearly all the PH3 expressing cells are found away from the ventricular surface. We examined the location of cells in S-phase by BrdU incorporation. In control embryos, BrdU-positive cells are located in the basal part of the ventricular zone (Figure 5M, N), whereas BrdU-positive cells in the mutant lose this distribution and are scattered throughout the ventricular zone (Figure 5O, P; white arrows indicate BrdU-positive cells located at the mutant ventricular surface). These results show that loss of Apc function disrupts the normal cell-cycle stage-specific apical/basal distribution of cerebral cortical progenitor nuclei

**Figure 6:**
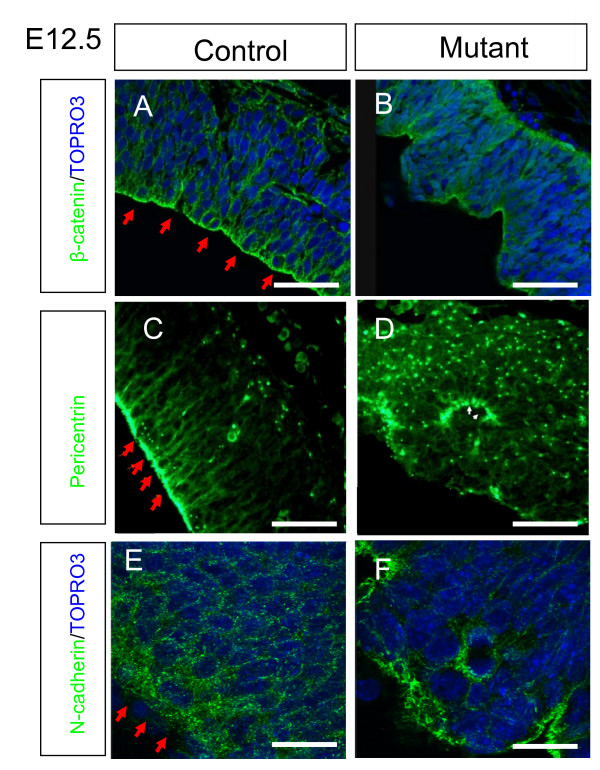
**Loss of cell polarity in mutant compared to control embryos at embryonic day (E)12.5**. **(A, B) **β-Catenin, **(C, D) **pericentrin, and **(E, F) **N-cadherin immunostaining (green) in control (A, C, E) and mutant embryos (B, D, F). Red arrows in (A, C) point to high levels of β-catenin and pericentrin, respectively, at the apical surface of the ventricular zone in control embryos; in the mutant (B, D) the apical localisation of both proteins is lost. (E) In controls, N-cadherin is distributed around cells, with particularly large amounts seen at the apical surface (red arrows); in the mutant (F) this pattern is altered, with many cells exhibiting much lower (in some cases undetectable) levels of N-cadherin. All sections are coronal and dorsal is up. Scale bars: (A-D) 100 μm; (E, F) 20 μm.

The disturbed apical/basal distribution of cell cycle phases prompted us to ask if other aspects of cell polarity were defective. During normal development, cells in the ventricular zone exhibit a polarised distribution of β-catenin, with the highest concentration found at the apical surface (Figure 6A, arrows). The centrosome-associated protein pericentrin [42] is also concentrated at the apical surface (Figure 6C, arrows). Following loss of Apc, this polarised distribution is lost, with β-catenin (Figure 6B) and pericentrin (Figure 6D) both being more evenly distributed throughout the cerebral cortex. In the control cerebral cortex the cell adhesion protein N-cadherin is found around the cell membrane, with particularly intense staining seen at the apical surface of the ventricular zone (Figure 6E). In the mutant the N-cadherin staining is much weaker at the cell membrane and distributed in scattered patches at the apical surface of the cerebral cortical ventricular zone (Figure 6F). Overall, proteins normally concentrated at the apical surface in the control become more diffusely distributed in the mutant, consistent with a disruption to the normal mechanisms that maintain apical polarity.

### β-Catenin is disregulated following loss of Apc

**Figure 7:**
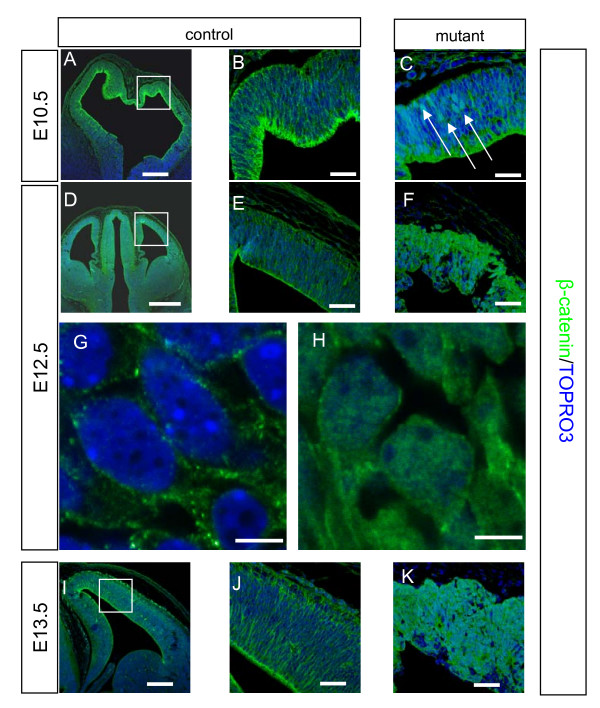
**(A-K) Progression of nuclear β-catenin (green) accumulation in the telencephalon of control (A, B, D, E, G, I, J) and mutant (C, F, H, K) embryos at embryonic day (E) 10.5 (A-C), E12.5 (D-H), and E13.5(I-K)**. **(A, D, I) **In the control, β-catenin staining is widespread throughout the cerebral cortex **(B, E, G, J) **Higher magnification images show that β-catenin is predominantly located at the cell surface and excluded from the nucleus. In contrast, the mutant has significant numbers of cells with β-catenin in the nucleus. These are first apparent at E10.5 **(C) **(white arrows indicate examples), becoming more pronounced at E12.5 **(F) **and E13.5 **(K)**. **(H) **A higher magnification image, showing a typical example of nuclear β-catenin at E12.5. All sections are coronal and dorsal is up. Scale bars (A, D) 250 μm; (B, C, E, F, J, K) 100 μm; (G, H) 5 μm.

Apc participates in a cytoplasmic protein complex with β-catenin that mediates its destruction in the absence of a Wnt signal. Loss of Apc might, therefore, be expected to result in increased levels of β-catenin but could also have other consequences for its cellular localisation. In the control cerebral cortex, β-catenin protein is concentrated at the cell surface, with particularly high levels apparent at the apical surface of the ventricular zone. This distribution is apparent throughout early corticogenesis from E10.5 to E13.5 (Figure 7A, B, D, E, G, I, J). In the mutant, β-catenin accumulates in the nucleus. This is first apparent at E10.5 (Figure 7C) and becomes more pronounced at E12.5 (Figure 7F, H) and E13.5 (Figure 7K). These results show that, in the mutant, nuclear β-catenin levels exceed those normally found in the developing cerebral cortex.

### Activation of Wnt/β-catenin target gene expression

**Figure 8:**
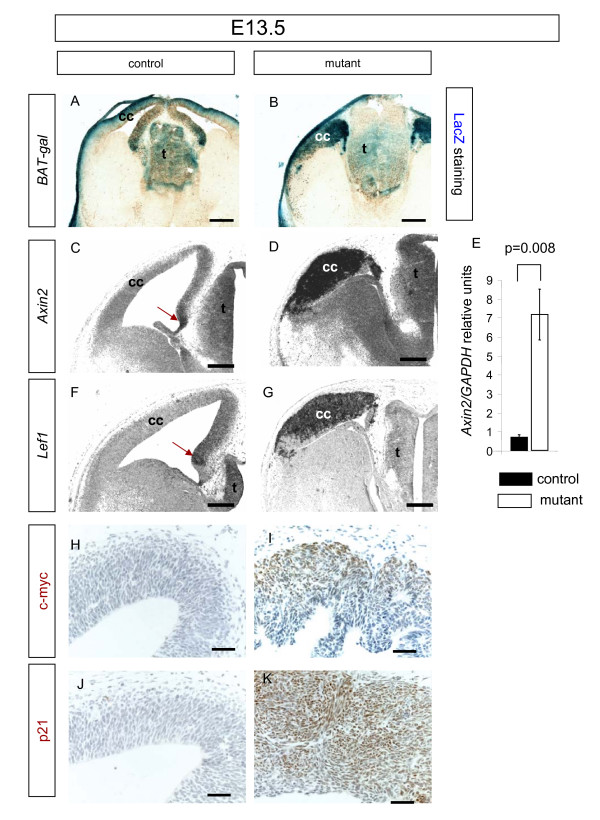
**Altered expression of β-catenin  target genes in mutant cerebral cortex**.  **(A, B)** X-gal staining (blue) for beta-galactosidase in the telencephalon of control (*Emx1*^*Cre*/+^*Apc*^*580S*/+^) and mutant (*Emx1*^*Cre*/+^*Apc*^*580S*/*580S*^) embryos at embryonic day (E)13.5 carrying the *BAT-gal *reporter transgene. X-gal staining in the cerebral cortex (cc) is less intense in the control (A) compared to the mutant (B). Brown immunostaining in (A, B) is for β-galactosidase. **(C, D, F, G) ***In situ *hybridisation for *Axin2 *(C, D) and *Lef1 *(F, G) transcripts (dark staining) in control (C, F) and mutant (D, G) forebrain at E13.5 presented as greyscale images. In controls, *Axin2 *expression is highest in the cortical hem (red arrow in (C)) and *Lef1 *expression is highest just adjacent to the hem (red arrow in (F)). In the mutant, expression of both *Axin2 *and *Lef1 *is high throughout the cerebral cortex. **(E) **qRT-PCR showing a large increase in the levels of *Axin2 *transcripts in the E13.5 cerebral cortex of mutant compared to control embryos. *P*-value calculated using Student's *t*-test is indicated above the bars. Error bars are standard error of the mean. The ubiquitous pale staining in (C-G) is non-specific background. **(H-K) **Immunostaining for c-myc and p21 (brown) in the telencephalon of control (H, J) and mutant embryos (I, K) at E13.5. The control cerebral cortex is negative for c-myc staining, but the mutant cerebral cortex has c-myc positive cells. P21 levels are also increased in the mutant. All sections are coronal and dorsal is up. Additional abbreviations: t, thalamus. Scale bars: (A, B) 400 μm; (C-G) 200 μm; (H-K) 100 μm.

We next investigated the possibility that increased levels of nuclear β-catenin in the mutant developing cerebral cortex enhance the transcription of Wnt/β-catenin target genes. The *BAT-gal *reporter transgene has seven fused Tcf/Lef-binding sites regulating the expression of β-galactosidase in response to β-catenin-specific activation [25]. We crossed the *BAT-gal *reporter allele onto our control and mutant animals in order to assess changes to β-catenin transcriptional activity resulting from loss of Apc function. The cerebral cortex reveals more intense LacZ staining in the mutant compared to the control (Figure 8B and 8A, respectively). These results suggest an over-activation of canonical Wnt/β-catenin signalling due to β-catenin stabilisation in the mutant. We next used *in situ *hybridisation and immunohistchemstry to examine the expression of endogenous genes *Axin2 *(*conductin*), *Lef1*, and *c-myc*, which are known to be up-regulated in response to Wnt/β-catenin signalling [43-45]. At E13.5 in controls, *Axin2 *and *Lef1 *are expressed most strongly in the medial dorsal telencephalon in the vicinity of the cortical hem, with relatively low levels of expression in the cerebral cortex itself (Figure 8C, F). In mutants at the same stage, high-level expression of *Axin2 *and *Lef1 *extends throughout the cerebral cortex (Figure 8D, G). qRT-PCR showed that there was an increase in levels of *Axin2 *transcripts in the mutants to about 10-fold those of the control (Figure 8E). At E13.5, immunostaining for c-myc shows that there is no expression of c-myc in the control (Figure 8H), whereas the mutant shows expression of this protein in the cerebral cortex (Figure 8I). Although there have been reports that the cell cycle regulator p21 is negatively regulated by Wnt/β-catenin signalling in colorectal cells or cell lines [46,47], we found that levels of p21 protein were actually elevated in the mutant cerebral cortex (compare Figure 5J and 5K). Levels of p21 have been found to increase when apoptosis is induced in APC-deficient cell lines [48] and increased apoptosis in our mutants (Figure 4) may be responsible for increased p21 levels. Alternatively, p21 expression in the cerebral cortex may not be regulated in the same way as in colorectal cells or cell lines. In conclusion, cells in the mutant cerebral cortex ectopically up-regulate the expression of genes known to be responsive to Wnt/β-catenin signalling, consistent with the idea that loss of Apc causes the activation of canonical Wnt signalling.

### Loss of Apc function causes cerebral cortical cells to adopt alternative fates

In the preceding section we demonstrate that loss of Apc function results in increased nuclear β-catenin and aberrant activation of Wnt/β-catenin target gene expression in the cerebral cortex. Wnt signalling is important for specifying cell identity in the embryonic central nervous system, so we next examined whether cerebral cortical cells alter their identity following loss of Apc.

**Figure 9:**
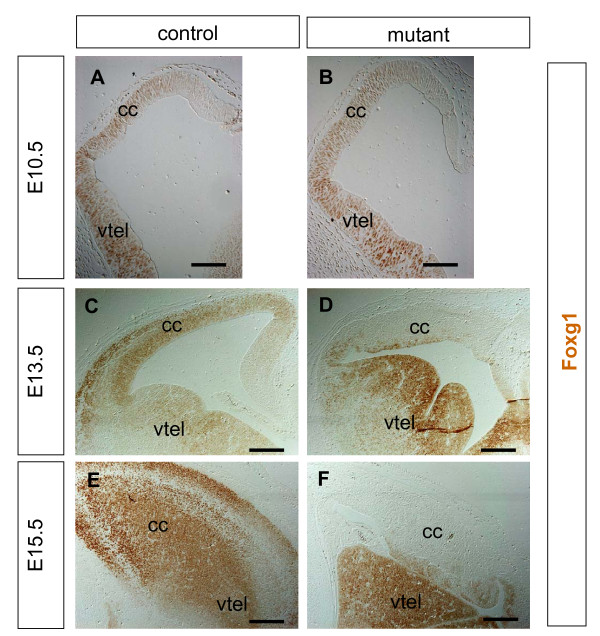
**Foxg1 expression (brown) in the telencephalon of control and mutant embryos at embryonic day (E)10.5, E13.5, and E15.5**. **(A, B) **E10.5 telencephalons of the control and the mutant have a similar pattern of expression in the future cerebral cortex (cc) and ventral telencephalon (vtel). **(C, D) **By E13.5 control (C) embryos retain expression in the cerebral cortex while mutants (D) exhibit very low levels of cerebral cortex expression, although expression in the ventral telencephalon is unaffected. (E, F) At E15.5, control embryos (E) expresses Foxg1 throughout the cerebral cortex whereas the mutant (F) has very low expression of Foxg1 in the cerebral cortex, although expression in the ventral telencephalon is unaffected. All sections are coronal, except (E, F), which are parasagittal, and dorsal is up. Scale bars: (A, B) 100 μm; (C-D) 200 μm.

**Figure 10:**
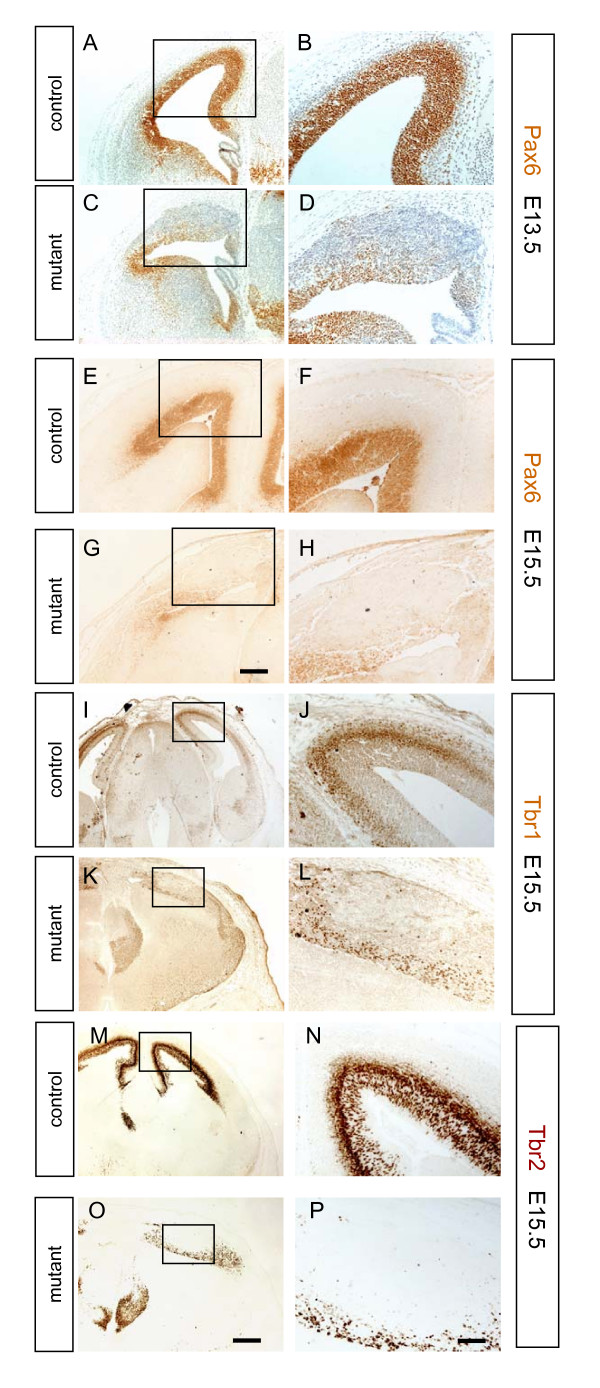
**Pax6, Tbr1, and Tbr2 expression in the cerebral cortex of control and mutant embryos**. **(A-H) **Pax6 immunohistochemistry of embryonic day (E)13.5 (A-D) and E15.5 brains (E-H). Expression of Pax6 is lower in the mutant both at E13.5 (C, D) and E15.5 (G, H) compared to control (A, B and E, F). **(I-L) **Expression pattern of Tbr1 (brown) in control and mutant embryos at E15.5. (I, J) In controls, Tbr1 is expressed in a laminar pattern in the cortical plate. (K, L) In the mutant, Tbr1 expression is reduced, with expressing cells concentrated at the ventricular surface. **(M-P) **Expression pattern of Tbr2 in contraol and mutant embryos at E15.5. (M, N) In controls, Tbr2 (brown) is expressed in a laminar pattern in the subventricular zone and in the cortical plate. (O, P) In the mutant, Tbr2 expression is reduced with expressing cells concentrated at the ventricular surface. Boxed areas in (A, C, E, G, I, K, M, O) are shown at higher magnification in (B, D, F, H, J, L, N, P). All sections are coronal and dorsal is up. Scale bars: (A, C, E, G) are to same scale with bar in (G) 200 μm; (I, K, M, O) are to same scale with bar in (O) 400 μm; (B, D, F, H, J, L, N, P) are to same scale with bar in (P) 100 μm.

During its normal development, the cerebral cortex is characterised by the expression of a number of transcription factors. These include the winged helix transcription factor Foxg1 [49,50], the homeodomain transcription factor Pax6 [51,52] and the T-box transcription factors Tbr1 and Tbr2 [30,53]. At E10.5, both control (Figure 9A) and mutant (Figure 9B) cerebral cortex express Foxg1. In controls, Foxg1 expression continues at later ages, with strong expression seen in the cerebral cortex at E13.5 (Figure 9C) and E15.5 (Figure 9E). In contrast, Foxg1 expression is severely reduced in the mutant cerebral cortex at E13.5 (Figure 9D) and E15.5 (Figure 9F) while expression in the mutant ventral telencephalon is unaffected. In controls at E13.5 (Figure 10A, B) and E15.5 (Figure 10E, F), Pax6 is expressed at high levels by cells in the ventricular zone of the cerebral cortex. In mutant embryos at E13.5 (Figure 10C, D) and E15.5 (Figure 10G, H), there are many fewer cells expressing Pax6. At E15.5, Tbr1 is expressed strongly by early born cortical neurons in the cortical plate of control embryos (Figure 10I, J) and Tbr2 is expressed by cells in the subventricular zone and cortical plate (Figure 10M, N). In mutant embryos there are fewer Tbr1- and Tbr2-expressing cells and these are scattered towards the ventricular surface of the mutant cerebral cortex (Figure 10K, L, O, P). Our finding that many cerebral cortical cells do not express molecular markers normally characteristic of this region prompted us to examine whether they may have adopted alternative fates.

**Figure 11:**
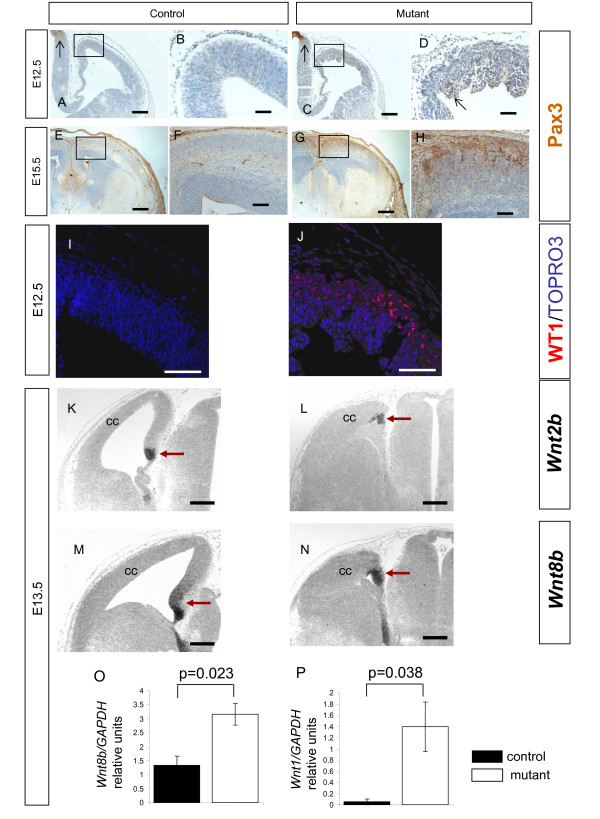
**Expression of *Pax3*, *Wt1*, *Wnt2b*, *Wnt8b*, and *Wnt1 *in the developing cerebral cortex of control and mutant embryos**. **(A-H) **Pax3 immunostaining in control (A, B, E, F) and mutant (C, D, G, H) embryos at embryonic day (E)12.5 (A-D) and E15.5 (E-H). At E12.5, Pax3 positive cells are present in the epithalamus of the control (A, black arrow) and the mutant (D, black arrow). The control cortex is free of Pax3 expression (B). There are a few Pax3 positive cells in the mutant cerebral cortex (D, black arrow). At E15.5, there is no expression of Pax3 by the control cortex (E, F). Pax3 expression is increased in the mutant cerebral cortex (G, H) and Pax3 immunopositive cells are located in upper layers of the cerebral cortex (H). **(I, J) **Immunostaining for Wt1 (red) in control (I) and mutant embryos (J) at E12.5. Wt1 expression is only seen in the mutant. **(K-N) ***In situ *hybridisation for *Wnt2b *(K, L) and *Wnt8b *(M, N) transcripts (dark staining) presented as greyscale images in control (K, M) and mutant forebrain (L, N) at E13.5. In control embryos, *Wnt2b *(K) and *Wnt8b *(M) expressionis largely restricted to the cortical hem region, with *Wnt8b *expression extending more dorsally into the medial telencephalon (red arrows). In mutant embryos, *Wnt2b *(L) and *Wnt8b *(N) expression is similarly restricted (red arrows) and does not extend into the cerebral cortex. The ubiquitous pale staining is non-specific background. **(O, P) **qRT-PCR of RNA extracted from E13.5 cerebral cortex of mutant compared to control embryos showing a modest increase in the levels of *Wnt8b *(O) and a massive increase in the levels of *Wnt1 *transcripts (P) relative to *GAPDH*. Student's *t*-test *p*-values are indicated above the bars. Error bars are standard error of the mean. All sections are coronal and dorsal is up. Abbreviations: cc, cerebral cortex. Scale bars: (A, C) 200 μm; (B, D, I, J) 50 μm; (E, G) 400 μm; (K-N) 200 μm.

We next examined the expression of genes that are not normally expressed in the developing telencephalon but are expressed in more posterior-dorsal regions of the brain where levels of Wnt/β-catenin signalling are normally higher than in the cerebral cortex. We found that the transcription factors Pax3 [54] and Wt1 [55] are expressed in the developing cerebral cortex of mutant embryos (Figure 11C, D, G, H, J) but are not detected in control embryos at the same ages (Figure 11A, B, E, F, I). We used qRT-PCR to show that *Wnt1 *[56-58] transcripts are easily detectable in mutant (but not in control) cerebral cortex at E13.5 (Figure 11P).

Cells in the mutant cerebral cortex exhibit gene expression patterns normally diagnostic of elevated Wnt/β-catenin signalling (Figure 8) in combination with reduced levels of the transcription factor Foxg1 (Figure 9). These molecular properties are characteristic of cells in the cortical hem region and presumptive hippocampus of the medial dorsal telencephalon [59-61], prompting us to speculate that cells in the mutant cerebral cortex had adopted the fate of this region. To test this hypothesis, we used *in situ *hybridisation to examine the expression of *Wnt2b*, which is normally expressed at high levels in the cortical hem region, and *Wnt8b*, which is normally expressed in the cortical hem region with expression extending dorsally into the presumptive hippocampus [59,62]. Contrary to our hypothesis, we found that neither *Wnt2b *(compare Figure 11K and 11L) nor *Wnt8b *(compare Figure 11M and 11N) were ectopically expressed in the mutant cerebral cortex (compare staining intensity in the cerebral cortex to that in the cortical hem/hippocampal region in Figure 11K–N). qRT-PCR on RNA extracted from control and mutant dorsal telencephalon revealed a twofold increase in *Wnt8b *expression (Figure 11O). As *Wnt8b *expression had not extended into the mutant cerebral cortex, this modest increase might be a local up-regulation of *Wnt8b *expression within the cortical hem region or reflect the reduced size of the mutant cerebral cortex relative to the cortical hem region. The *Emx1*^*Cre *^allele we used to inactivate *Apc *is not active in the cortical hem itself (Figure 1) [33], so it is not surprising that the molecular characteristics of the cortical hem are not affected in *Apc *mutants (compare cortical hem region expression of *Axin2 *and *Lef1 *expression in Figure 8 and *Wnt2b *and *Wnt8b *expression in Figure 11 between control and mutant embryos). The mutant cerebral cortex does not, therefore, correspond to a lateral expansion of cortical hem or hippocampal identity.

The loss of Apc function therefore causes dorsal telencephalic cells to adopt molecular characteristics normally associated with more posterior-dorsal parts of the central nervous system.

### Shh signalling is not upregulated following loss of Apc

**Figure 12:**
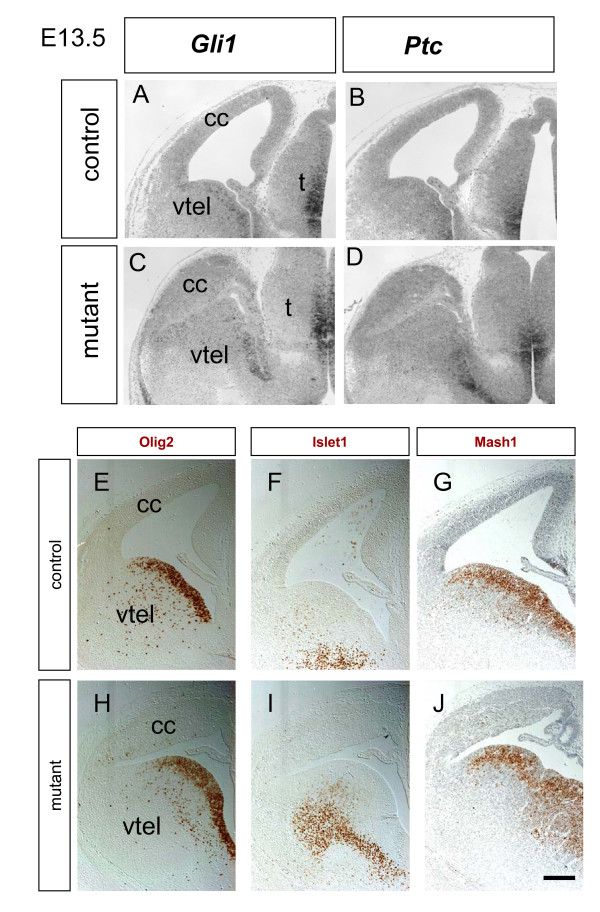
**Expression of *Gli1*, *Ptc*, *Olig2*, *Islet1*, and *Mash1 *in the forebrain of control and mutant embryos at embryonic day (E)13.5**. **(A-D) ***In situ *hybridisation for *Gli1 *(A, C) and *Ptc *(B, D) transcripts (dark staining) in control (A, B) and mutant (C, D) presented as greyscale images. *Gli1 *and *Ptc *are expressed at similarly low levels in the cerebral cortex (cc) of control embryos and mutant embryos. Both transcripts are expressed at similarly high levels in the thalamus (t) of control and mutant embryos. The ubiquitous pale staining is non-specific background. **(E-J) **Immunohistochemistry for Olig2 (E, H), Islet1 (F, I), and Mash1 (G, J) proteins (brown) in control (E-G) and mutant forebrain (H-J) at E13.5. In both control and mutant embryos, Olig2, Islet1, and Mash1 are strongly expressed in ventral telencephalic (vtel) domains but not in the cerebral cortex. All images to same scale, bar in (J) 200 μm.

We next investigated the possibility that the disorganisation we observe in the *Apc *mutant cerebral cortex might be a contributory factor to altering the molecular identity of its cells. It has been reported that disrupting adherens junctions in *αE-catenin *loss-of-function mutants results in up-regulation of Shh signalling via a mechanism independent of altered Wnt/β-catenin signalling [63,64]. As β-catenin is a component of adherens junctions, the disregulation of β-catenin function in the *Apc *mutants could conceivably produce disruption to normal adherens junctions, causing up-regulation of Shh signalling, which could in turn affect cell identity. The *αE-catenin *mutant cerebral cortex shares some features in common with the *Apc *mutant – for example, increased numbers of mitoses away from the apical surface of the ventricular zone – although they differ in other respects – for example, the *αE-catenin *mutant exhibits increased cell proliferation whereas the *Apc *mutant does not. To test the possibility that increased Shh signalling might be a contributory factor to our *Apc *mutant phenotype, we examined the expression of the Shh transcriptional targets, the transcription factors *Gli1 *and *Olig2*, and the Shh receptor *Ptc *[65-69]. We found that expression of *Gli1*, *Ptc *and *Olig2 *in the cerebral cortex at E13.5 is below our limits of detection in both control and mutant cerebral cortex, and far below the levels seen in the thalamus (compare Figure 12A, B, E to 12C, D, H). This indicates that Shh signalling is not up-regulated in the cerebral cortex following loss of Apc function. Shh signalling is associated with adoption of ventral fate in the central nervous system and we used immunohistochemistry to examine expression of the ventrally expressed transcription factors Islet1 and Mash1 [70-72]. We found no evidence for ectopic expression of these ventral markers in the cerebral cortex of the mutant at E13.5 (compare Figure 12F, G to 12I, J). It is, therefore, extremely unlikely that aberrant activation of Shh signalling can explain the *Apc *cerebral cortical loss-of-function phenotype.

## Discussion and conclusion

In this study we have examined the consequences of removing Apc protein from the developing mouse cerebral cortex after E9.5, an age by which it has already acquired molecular characteristics of the cerebral cortex. Apc is a multifunctional protein with roles in cell proliferation, differentiation, migration, and apoptosis [73], so it is perhaps not surprising that we find disruption of a multitude of developmental processes in *Apc *mutants.

### Disruption to β-catenin function in the *Apc *mutant

It is likely that many of the defects observed in conditional *Apc *mutants stem from a disregulation of β-catenin function, although loss of Apc function may also have consequences independent of β-catenin. The earliest abnormality we detected was an increase in nuclear β-catenin at E10.5 followed by up-regulation in the expression of β-catenin/TCF/Lef transcriptional targets, including the *BAT-gal *reporter transgene and endogenous targets *c-myc*, *Lef1 *and *Axin2*. A key role of Apc is to mediate the destruction of β-catenin, a process that is blocked when the cell receives a Wnt signal [17]. Loss of Apc function might, therefore, be expected to produce similar effects to Wnt signalling and/or β-catenin stabilisation. Transgenic mice in which a stabilised β-catenin mutant protein was expressed in central nervous system progenitor cells under the control of a nestin promoter had shown dramatically enlarged cerebral cortex surface area, although lamination was not affected [74,75]. Increased brain size was explained by expansion of the progenitor pool due to cells re-entering the cell cycle instead of differentiating. In contrast, our *Apc *mutants exhibit a massive reduction in the size of the cerebral cortex, resulting from a combination of reduced proliferative pool size and increased cell death by apoptosis, and lamination is disrupted. These differences can likely be explained by differences in the timing of β-catenin stabilisation since the timing of Wnt/β-catenin signalling is known to be critical for neural cell fate decisions, and stabilising β-catenin during early cerebral cortical development promotes proliferation while at later stages it promotes neural differentiation [76-80]. The nestin promoter used by Chenn and Walsh results in expression of stabilised β-catenin in neural precursors [81] before they become committed to a cerebral cortical fate and start to express *Emx1 *at E9.5, whereas in our model nuclear β-catenin is first detected at E10.5 after the onset of *Emx1 *expression.

The magnitude of Wnt/β-catenin signalling experienced by cells is tightly regulated during development by the combined action of Wnt proteins and their antagonists. A number of Wnt proteins are secreted by cells in the cerebral cortex and by the cortical hem in the medial telencephalon [59,82]. Wnt antagonists – for example, Sfrp and Dickkopf proteins – are expressed in complex spatiotemporal patterns during cerebral cortical development [83-87]. By disrupting Apc we short-circuited these regulatory mechanisms as β-catenin is stabilised, and translocated to the nucleus, regardless of the presence of a Wnt signal. This results in the expression of Wnt/β-catenin target genes at higher levels than normally experienced by cerebral cortical cells. The severe consequences for cerebral cortical development emphasise the importance of maintaining the correct levels of Wnt/β-catenin signalling for the acquisition of the correct fate by cells in the developing cerebral cortex. One of our major findings is that many cells in the *Apc *mutant stop expressing genes normally expressed by cerebral cortex and start to express genes normally expressed elsewhere, indicating altered identity (see below). Critically, these changes occur after the cerebral cortex has become committed to its fate as *Apc *is inactivated after the expression of *Emx1*. Our results indicate, therefore, that acquisition of cerebral cortical neural fates is reversible. An interesting possibility is that Apc is required to stabilise the acquisition of cerebral cortical cell fates and that loss of Apc allows cells to redirect to alternative fates.

### Apc and cell polarity

A striking feature of *Apc *mutants is the disorganisation of the cerebral cortex. In wild-type embryos the nuclei of cells undergoing M-phase line up along the ventricular surface and then travel deeper into the ventricular zone before entering S-phase. When Apc is inactivated the molecular and cellular properties of the ventricular zone are severely affected. Pericentrin, β-catenin, and N-cadherin proteins are normally concentrated at the apical surface of the ventricular zone and this normally polarised organisation is lost in the *Apc *mutants. The normal positioning of M-phase nuclei at the apical surface and S-phase nuclei away from the apical surface is lost in the mutants. This suggests that Apc is required for the establishment or maintenance of apical identity and indeed we observed particularly high levels of Apc protein at the apical surface of the ventricular zone from E11.5 to E14.5. Apc might function by polarising the response to Wnt signalling within the cell and/or by tethering proteins (including pericentrin, β-catenin, and N-cadherin) and regulating their polarised distribution within the cell. N-cadherin is a cell adhesion molecule that functions by coupling to the cytoplasmic catenin proteins (including β-catenin). Developing cerebral cortex lacking N-cadherin [88] exhibits many defects that resemble those seen when *Apc *is inactivated (present study), including disrupted interkinetic nuclear migration, loss of cell polarity, and disorganisation of cerebral cortical layers. A conditional β-catenin loss of function mutant [80] also exhibited disrupted interkinetic nuclear migration and cerebral cortical disorganisation reminiscent of both our *Apc *mutant and the N-cadherin mutant. This provides support for the hypothesis that *Apc*, *β-catenin*, and *N-cadherin *co-operate genetically during normal cerebral cortical development and that loss of Apc function causes loss of N-cadherin and β-catenin functions. Mechanistically, this could occur at the level of disruption to cytoskeletal protein interactions and/or by means of altered gene expression in the *Apc *mutant. In the developing limb bud, nuclear β-catenin down-regulates expression of cadherin [89] and the altered N-cadherin distribution seen in our mutant could be caused by a down-regulation in gene expression caused by elevated levels of nuclear β-catenin.

### Apc and cell identity

The position of cells in the developing cerebral cortex is normally tightly regulated and it is possible that the failure of cells to adopt their correct positions in the *Apc *mutant might affect their molecular environment and this might cause them to adopt alternative fates. It has recently been shown that conditional deletion of the small Rho-GTPase *cdc42 *in the developing cerebral cortex disrupts interkinetic nuclear migration and cell polarity, with fewer cells undergoing mitosis at the apical surface of the ventricular zone [90]. The corresponding increase in basally located mitosis correlates with an increase in the number of cells acquiring the fate of basal progenitors and expressing the transcription factor Tbr2. Although the *cdc42 *mutant resembles our *Apc *mutant with respect to the shift in balance from apical to basal mitosis, we actually see a decrease in the numbers of Tbr2 expressing cells (Figure 10). Executing mitosis away from the ventricular surface in *Apc *mutants is, therefore, not sufficient to direct cells to a basal cerebral cortical Tbr2 expressing fate. The *Apc *mutant is not the simplest model in which to study correlations between basal mitosis and post-mitotic Tbr2 expression as the loss of cerebral cortical identity found in *Apc *mutants might independently contribute to the reduction in the numbers of cells expressing Tbr2. It is extremely unlikely that the alterations to cell identity in the *Apc *mutant stem from an ectopic upregualtion of the Shh pathway as cells in the *Apc *mutant cerebral cortex do not ectopically express the Shh target genes *Gli1*, *Ptc*, or *Olig2*.

There is accumulating evidence that high levels of Wnt/β-catenin signalling during early vertebrate development promote posterior neural cell fates at the expense of anterior neural cell fates and that the formation of anterior head structures requires the active suppression of Wnt/β-catenin signalling [91-94]. Wnt/β-catenin signalling is also tightly regulated along the dorsal-ventral axis such that higher levels of Wnt/β-catenin activity generally map to dorsal central nervous system structures [25].

The failure of the *Apc *mutant cerebral cortex to express transcription factors Pax6, Foxg1, Tbr1, and Tbr2 indicates that they lose their cerebral cortical identity, raising the question of what identity they acquire. One possibility is that ectopic activation of Wnt/β-catenin signalling in the cerebral cortex of the *Apc *mutant might redirect cerebral cortical cells to adopt fates normally associated with more posterior-dorsal central nervous system structures (such as dorsal diencephalon, dorsal midbrain, dorsal hindbrain and dorsal spinal cord) where Wnt/β-catenin signalling is high. To test this hypothesis, we examined the expression of three genes (*Pax3*, *Wnt1*, and *Wt1*) normally expressed in posterior/dorsal brain structures. Pax3 is normally expressed dorsally in the epithalamus, in the ventricular zone at the mesencephalic-rhombencephalic border, in the dorsal part of the ventricular zone and the roof plate of the medulla oblongata, and the dorsal spinal cord [54,95]. *Wnt1 *expression is normally restricted to the dorsal and ventral midline of the midbrain and caudal diencephalons, a narrow ring rostral to the midbrain-hindbrain junction and the roof plate of the spinal cord [56-58]. The transcription factor Pax3 can regulate the expression of *Wnt1 *by binding to DNA sequences in its promoter [96], which may account for the similarities in their expression domains. Wt1 is normally expressed in the roofplate of the midbrain and in the spinal cord [55]. Conversely, *Gli1*, *Ptc*, *Mash1*, *Olig2*, *and Islet1 *are normally expressed in ventral regions of the central nervous system where Wnt/β-catenin signalling is generally low [25]. Our finding that ectopic expression of *Pax3*, *Wnt1*, and *Wt1*, but not *Gli1*, *Ptc*, *Mash1*, *Olig2*, or *Islet1*, occurs in the *Apc *mutant cerebral cortex is consistent with the idea that loss of Apc simulates enhanced Wnt/β-catenin signalling and pushes cerebral cortical cells towards posterior-dorsal, but not ventral, fates. Following this reasoning, it might be predicted that the *Apc *mutant cerebral cortex would ectopically express molecular characteristics of the cortical hem region and presumptive hippocampus, dorsal-medial telencephalic structures adjacent to the cerebral cortex and characterised by high levels of Wnt/β-catenin signalling and expression of Wnt genes, including *Wnt2b *and *Wnt8b *and low expression of *Foxg1 *[25,59,60]. However, we found no ectopic expression of *Wnt2b *or *Wnt8b *in the *Apc *mutant cerebral cortex, raising the possibility that some factor in the cerebral cortex in the *Apc *mutant somehow protects it from lateral to medial re-patterning and acquiring cortical hem region identity. Foxg1 [50,60] is a good candidate for this factor. Conditional *Foxg1 *mutants in which *Foxg1 *is deleted after E13.5 do not exhibit an enlarged cortical hem region [97,98], although *Foxg1*^-/- ^null embryos do exhibit large scale lateral to medial re-patterning of the telencephalon [61]. Our *Apc *mutants express Foxg1 normally at E10.5 but subsequently lose expression in the cerebral cortex with almost none left at E13.5. One possibility, therefore, is that early expression of Foxg1 is sufficient to prevent the acquisition of dorsal-medial telencephalic fate by the more lateral cerebral cortex. Other possibilities are that the levels of β-catenin transcriptional activation in our mutants are not appropriate to specify cortical hem region identity on the cerebral cortex or that there is a requirement for Apc itself in this process.

### Apc and cell differentiation

The activation of Wnt/β-catenin mediated gene expression varies during the division and maturation of cerebral cortical cells [20]. Proliferating cells in the ventricular zone exhibit high levels of β-catenin signalling, which is down-regulated as they exit the cell cycle and start their radial migration to the cortical plate and then up-regulated as they reach their destinations in the cortical plate [20]. Loss- and gain-of-function experiments have identified roles for β-catenin in neuronal differentiation as well as proliferation [20,76-80]. In this study we identified an up-regulation of *Apc*/Apc during the early development of the cerebral cortex that coincides with increasing numbers of post-mitotic cortical plate cells expressing high levels of Apc. This raised the possibility that Apc is required for the proper differentiation of cells within the cortical plate. Consistent with this idea, we showed that the transcription factors Tbr1 and Tbr2 do not exhibit their normal laminar distribution in the *Apc *mutant cerebral cortex. The failure of mutant cells to occupy their normal locations indicates defects in cell adhesion or migration consistent with previously described roles for Apc [99,100].

We found that the *Apc *mutant cerebral cortex has lost many aspects of its molecular identity by E13.5. The severe disruption to multiple aspects of cerebral cortical development we see in *Apc *mutants by E13.5 makes it difficult to address the primary function(s) of Apc in the differentiation of more mature cell types generated at later stages because it becomes increasingly difficult to disentangle the primary consequences of Apc disruption from the secondary consequences of major tissue insult and re-specification. In order to directly address the primary functions of Apc in the differentiation of cerebral cortical cells, it will be necessary to manipulate Apc function in these cells at later stages of development than in the current *Emx1*^*Cre*/+^*Apc*^*580S*/*580S *^transgenic model.

## Abbreviations

Apc: Adenomatous polyposis coli; BrdU: bromodeoxyuridine; E: embryonic day; FACS: fluorescence activated cell scanning; IdU: iododeoxyuridine; PBS: phosphate buffered saline; PH3: phosphohistone H3; qRT-PCR: quantitative reverse transcriptase PCR; TUNEL: terminal deoxynucleotidyl nick end labelling.

## Competing interests

The authors declare that they have no competing interests.

## Authors' contributions

UI carried out the experiments, participated in the design of the study, and helped draft the manuscript. YC performed the Apc immunohistochemistry on sections. JOM, DJP, and TP conceived of the study, participated in the design of the study, and helped draft the manuscript. All authors have read and approved the final manuscript.
